# Fabrication and Intracellular Delivery of Doxorubicin/Carbonate Apatite Nanocomposites: Effect on Growth Retardation of Established Colon Tumor

**DOI:** 10.1371/journal.pone.0060428

**Published:** 2013-04-16

**Authors:** Sharif Hossain, Hirofumi Yamamoto, Ezharul Hoque Chowdhury, Xin Wu, Hajime Hirose, Amranul Haque, Yuichiro Doki, Masaki Mori, Toshihiro Akaike

**Affiliations:** 1 Department of Biomolecular Engineering, Graduate School of Bioscience and Biotechnology, Tokyo Institute of Technology, Midori-ku, Yokohama, Japan; 2 Department of Surgery, Gastroenterological Surgery, Graduate School of Medicine, Osaka University, Suita-City, Osaka, Japan; 3 Jeffrey Cheah School of Medicine and Health Sciences, Monash University, Sunway Campus, Malaysia; 4 Department of Biomedical Engineering, University of California Davis, Davis, California, United States of America; Case Western Reserve University, United States of America

## Abstract

In continuing search for effective treatments of cancer, the emerging model aims at efficient intracellular delivery of therapeutics into tumor cells in order to increase the drug concentration. However, the implementation of this strategy suffers from inefficient cellular uptake and drug resistance. Therefore, pH-sensitive nanosystems have recently been developed to target slightly acidic extracellular pH environment of solid tumors. The pH targeting approach is regarded as a more general strategy than conventional specific tumor cell surface targeting approaches, because the acidic tumor microclimate is most common in solid tumors. When nanosystems are combined with triggered release mechanisms in endosomal or lysosomal acidic pH along with endosomolytic capability, the nanocarriers demonstrated to overcome multidrug resistance of various tumors. Here, novel pH sensitive carbonate apatite has been fabricated to efficiently deliver anticancer drug Doxorubicin (DOX) to cancer cells, by virtue of its pH sensitivity being quite unstable under an acidic condition in endosomes and the desirable size of the resulting apatite-DOX for efficient cellular uptake as revealed by scanning electron microscopy. Florescence microscopy and flow cytometry analyses demonstrated significant uptake of drug (92%) when complexed with apatite nanoparticles. *In vitro* chemosensitivity assay revealed that apatite-DOX nanoparticles executed high cytotoxicity in several human cancer cell lines compared to free drugs and consequently apatite-DOX-facilitated enhanced tumor inhibitory effect was observed in colorectal tumor model within BALB/cA nude mice, thereby shedding light on their potential applications in cancer therapy.

## Introduction

Contemporary cancer therapy, particularly with respect to drug delivery, has begun an evolution from traditional methodology. Part of this change is based on the need to increase the therapeutic index of chemotherapy drugs. Since the central problem in conventional cancer chemotherapy is the severe toxic side effects of anticancer drugs on healthy tissues and invariably the side effects impose dose reduction, treatment delay, or discontinuation of therapy [1], therefore delivery of specific drugs into the cancer cells to achieve high cytotoxicity with minimal side effect is a desirable objective for offering a potential approach for cancer therapy. To achieve these goals, the current focus is on the development of novel carriers with prolonged blood circulation time for both existing and incoming new drugs in order to achieve high efficacy in killing cancer cells and minimizing the side effects, while defining better therapeutic targets in various cancer cells [2], [3]. Consequently, in the past decade, nanoparticle-based drug delivery systems have shown exciting efficacy for cancer treatments due to their improved pharmacokinetics and biodistribution profiles via the enhanced permeability and retention (EPR) effect [4]–[6]. However, the EPR effect can only enhance the accumulation of nanoparticles in tumor tissues; the poor cellular internalization as well as insufficient intracellular drug release always limits the dosages of anticancer drugs to the level below the therapeutic window, which hampers the efficacy of cancer chemotherapy [7], [8]. To address the challenges, environment-responsive delivery systems have been attempted to improve the drug bioavailability [9], [10]. Of these stimuli, pH-responsiveness is the most frequently used, as pH values in different tissues and cellular compartments vary tremendously. A more advanced approach to efficient cytoplasmic drug release to cancer cells is to use nanoparticles as drug vehicles with lysosomal pH-triggered fast release kinetics for the associated drugs overcoming multidrug resistance (MDR) [11], [12]. In spite of the design of various drug carriers based on liposomal [13], [14] and polymeric formulations [15]–[19], an ideal system in terms of stability and subsequently proper intracellular delivery to the target cells is still lacking. Thus simple preparation of an efficient intracellular stimuli responsive drug delivery system is highly expected.

Doxorubicin (DOX), one of the most commonly used antitumor drugs, has a broad spectrum of antitumor properties in therapy against hematopoietic malignancies and solid tumors [20], [21]. However, DOX may produce free radicals, leading to serious side effects especially cardiomyopathy en route to congestive heart failure [20], [22]–[23]. Among the synthetic inorganic carriers, pH-sensitive nanoparticles of carbonate apatite being formed in a supersaturated solution of calcium, phosphate and carbonate ions have been getting much attention by virtue of their biodegradability and resemblance to body hard tissue components. Moreover, owing to its heterogeneous charge distribution and several fascinating properties such as ability of preventing crystal growth for generation of nanoscale particles as needed for efficient endocytosis, and fast dissolution kinetics in endosomal acidic compartments to facilitate the release of therapeutics from the particles and endosomes, carbonate apatite has been proven as the potential tool for the delivery of different therapeutics [24]–[33].

Therefore, recently pH sensitive carbonate apatite-DOX complexes were successfully fabricated for the first time for intracellular delivery of anticancer drug DOX since the carbonate apatite being highly stable in typical physiological pH could easily be dissolved in endosomal acidic pH following endocytosis, thus quickly releasing the associated drugs in cytoplasm for effective therapeutic action. We showed for the first time that the inorganic crystal had characteristics of nano-scale effective for passive targetting, enhanced cellular uptake and quick release of DOX in response to endosomal low pH, resulting in enhanced colorectal tumor inhibitory effects both *in vitro* and *in vivo*. The extraordinary proliferation inhibitory effect in a number of cancer cell lines was achieved by virtue of its pH sensitivity resulting in quick release of the drugs from the carrier and probably overcoming the MDR and getting advantages over different slow release carrier systems. Thus, we propose a novel nano-based pH targeting therapeutic strategy against malignancies, which is highly promising for preclinical and clinical cancer therapy.

## Materials and Methods

### Reagents, cell lines and mice

The human colon cancer cell lines SW480, HCT116 and the human ovarian cancer NIH:OVCAR-3 cell line were obtained from the American Type Culture Collection (Manassas, VA) and the Cell Resource Center for Biomedical Research, Institute of Development, Aging and Cancer, Tohoku University (Sendai, Japan) respectively. Dimethylsulfoxide (DMSO), MTT (3-(4,5-Dimethylthiazol-2-yl)-2,5-diphenyl tetrazolium bromide) were purchased from Sigma. Penicillin-streptomycin and FBS were purchased from Gibco BRL. Doxorubicin hydrochloride (DOX) and 7 weeks old Female BALB/cA nude mice were purchased from WAKO, Japan and CLEA Japan (Tokyo, Japan) respectively. sw480, HCT116 and NIH:OVCAR-3 cell lines were cultured on tissure culture dishes in Dulbecco's modified Eagle's medium (DMEM, Gibco BRL) and RPMI 1640 medium (Gibco BRL) respectively supplemented with 10% fetal bovine serum (FBS), 50 µg/ml penicillin, 50 µg/ml streptomycin, 100 µg/ml neomycin at 37°C in a humidified 5% CO2-containing atmosphere.

### Apatite-DOX complex formation and subsequent delivery to cancer cells

Cells from the exponentially growth phase were seeded at 40,000 cells per well into 24-well plates the day before drug delivery. 1 to 5 μl of 1 M CaCl_2_ was mixed with 0 to 80 μM of DOX and added to 1 ml of fresh serum free bicarbonate (44 mM)-buffered DMEM medium (pH 7.5), followed by incubation at 37°C for 30 min, for complete generation of apatite-DOX particles. Then the particle suspensions were centrifuged at 15000 rpm for 3 min and the pellets were washed three times to remove the free unbound DOX. Medium with generated DOX-containing particles (DOX load 10–1000 nM) was added with 10% FBS to the rinsed cells.

### Evaluation of the DOX loading onto carbonate apatite by florescence intensity and high performance liquid chromatography (HPLC)

Following generation of carbonate apatite-DOX particles using 3 mM Ca^2+^ and 20∼80 µM of DOX, the resulting pellet was dissolved in 100 µl of 10 mM EDTA-PBS, quantified for the florescence intensity by a spectrophotometer (λex 485 nm, λem 595 nm). The calibration curve was made by plotting the value of control free DOX sample (1, 10, 100 nM & 1, 10, 50, 100 µM) in PBS. In case of HPLC, Daunorubicin was added as an internal standard in 50 μl sample, which was injected into an HPLC apparatus (HP1100, Agilent, USA) equipped with an Inertsil WP300 C18 column (GL Science, Japan) at 40°C and a fluorescence detector (excitation wavelength, 470 nm; emission wavelength, 585 nm). The mobile phase was a mixture of formic acid buffer (0.28M, pH 3.55)/acetone/2-propanol [65∶30∶5 (v/v)] and the flow rate was 1.0 ml/min. Quantification was based on peak area ratio of DOX to the internal standard.

### Characterization of generated particle by attenuated total reflection-fourier transform infra red (ATR-FTIR) spectroscopy

Following generation of carbonate apatite as described above, using 3 mM Ca^2+^ with or without DOX, precipitated particles were lyophilized after centrifugation and washing with distilled deionized water. Fourier transform-infrared spectroscopy (FT-IR) of the apatite particles was performed using FT/IR-230, JASCO. The samples were ground in a mortar and approximately 1 mg was thoroughly mixed with 300 mg of ground spectroscopic grade KBr. Transparent pellets were prepared in a KBr die with an applied load of 8000 kg, under a vacuum of 0.5 Torr.

### Particle size measurements

The distribution of particle size was measured using FPAR-1000 fiberoptics particle analyzer (Otsuka Electronics, Osaka, Japan) equipped with a 660 nm diode laser. The measurement was carried out by a scattering angle of 90° at 25°C. The size distribution was obtained by CONTIN method.

### Observation of apatite-DOX complexes with transmission electron microscope (TEM) & atomic force microscopy (AFM)

Apatite-DOX complexes were observed under H-7500 transmission electron microscope (Hitachi, Tokyo, Japan) at an acceleration voltage of 80 kV. After loading of the particles on the copper grid with collodion membrane, the grid was dried in the air. Moreover, a scanning probe microscope (SPM-9500, Shimadzu, Japan) in dynamic mode, equipped with a micro cantilever (OMCL-AC160TS-C2, Olympus, Japan) was used to analyze the morphology and size of the carbonate apatite nano-particles.

### Estimation of the dissolution of carbonate apatite particles in acidic solution

300 ml of apatite-DOX complex suspension was prepared from DMEM containing 40 µM of DOX and 3 mM of exogenously added CaCl_2_ followed by incubation for 30 min at 37°C in a water bath. The pH of the suspension was decreased by adding 1 N HCl and 1 ml of the suspension at each pH was taken. Optical density at 320 nm of the collected sample was measured with SmartSpec™ 3000 spectrophotometer (Bio-Rad).

### Uptake behaviour of carbonate apatite by scanning electron microscope (SEM)

The HCT116 cells were uniformly seeded (3×10^5^/dish) into 35 mm dishes the day before delivery of carbonate apatite particles. At 45 min and 90 min after addition of apatite particles, cells were fixed in 2.5% glutaraldehyde for 90 min and in 1% osmium tetroxide for 1 hr at 4°C, dehydrated in graded ethanol, dried in desiccators overnight. Sputter-coated with an osmium layer (Neoc-cs, Meiwa, Japan), they were observed by a high resolution SEM (S-800, Hitachi, Japan).

### Cellular uptake of apatite-DOX particles by florescence microscopy and quantitative analysis by in vitro flow cytometry

Free DOX or DOX-loaded apatite particles (200 nM loaded DOX equivalent) were added onto the cells with 10% serum and kept for 4 h in the incubator for cellular internalization. Extracellular particles were removed by EDTA prior to observation of the cells by a fluorescence microscope (Olympus-IX71). For quantitative analysis by flow cytometry, NIH:OVCAR-3 ovarian cancer cells (0.5×10^6^ cells/dish) were seeded in a 60 mm tissue culture dish on the day before intracellular delivery and incubated overnight. 5 ml of apatite-DOX particles suspension prepared with 200 nM DOX concentration in medium was introduced to the cells and incubated for 4 h. The cells were then trypsinized, washed three times with PBS solution and then fixed with 10% formaldehyde. After filtering through a nylon mesh, cell fluorescence was measured by flow cytometer (FACSCAN, Becton Dickinson).

### *In vitro* DOX release from pH-sensitive carbonate apatite

Following generation of carbonate apatite-DOX particles using 3 mM Ca^2+^ and 20∼80 µM of DOX, the resulting pellet was dissolved in 2 ml of phosphate buffer saline (PBS, pH 7.4) or sodium acetate buffer (pH 5.5) at 37°C with a continuous shaking at 80 rpm. At given times, the solution was removed and the pH-dependant DOX release profiles were determined by measuring the UV – vis absorbance at 485 nm.

### Chemosensitivity assay

The cytotoxic effect of free DOX or apatite-DOX on the cells was determined by the MTT (3-(4,5-dimethylthiazol-2-yl)-2,5-diphenyltetrazolium bromide) assay. Briefly, Following incubation of free DOX or apatite-DOX complexes with SW480, HCT116 and NIH:OVCAR-3 cancer cells for 0∼72 h, 30 μl of MTT solution (5 mg/ml) was added to each well and incubated for 4 h at 37°C. 0.5 ml of DMSO was added after removal of media. After resolving crystals and incubating for 5 min at 37°C, absorbance was measured in a micro plate reader at 570 nm with a reference wavelength of 630 nm. Cell viabilities were normalized to the absorbance of non-treated cells.

### *In vivo* evaluation of antitumor activity of apatite-DOX complex

The *in vivo* efficacy of the apatite-DOX was assessed in female BALB/cA nude mice. This study was carried out in strict accordance with the recommendations in the Guide for the Care and Use of Laboratory Animals of Graduate School of Medicine, Osaka University. The protocol was approved by the Committee on the Ethics of Animal Experiments of Osaka University (Permit Number: 23-023-0). All surgery was performed under sodium pentobarbital anesthesia, and all efforts were made to minimize suffering. Seven-week-old BALB/cA female mice were used for tumor regression study. Human colorectal cancer HCT116 cells were inoculated s.c. in the both left and right flanks of the mice to generate the tumor. After the tumor induction when the diameter of tumors reached φ5 mm, tumor bearing animals were separated and divided randomly into three test groups (Control, Free-DOX, Apatite-DOX) consisting of three mice per group. Free-DOX at dose of 0.33 mg/kg/day or apatite-DOX containing the same amount of DOX was administered by intravenous injection into the tail vein on days 0,1,2,7,8,9,14,15, and 16. The control group received a single intravenous injection of PBS (pH 7.4). The antitumor activity was evaluated in terms of the tumor size (n = 6), which was estimated by the following equation: V =  (a)×(b)^2^/2, where (a) and (b) represent major and minor axes of the tumor, respectively.

## Results and Discussion

### Generation and estimation of DOX loading on apatite-DOX particles

After generation of carbonate apatite-DOX nanoparticles, the turbidity analysis of particle suspension at 320 nm demonstrated that the growth or formation of the particles following incubation of HCO_3_^−^−buffered DMEM (pH 7.5) containing a fixed concentration of Ca^2+^ (3 mM) and variable concentrations of DOX (0 to 80 µM) for a period of 30 min at 37°C, increased almost proportionately with an increase of DOX concentrations, suggesting that DOX might bind to the growing apatite particles resulting in the acceleration of the growth (Data not shown). Additionally, florescence analysis of the dissolved apatite-DOX particles proved the incorporation of DOX into apatite particles. Loaded DOX in carbonate apatite was measured either by florescence intensity of DOX using a spectrophotometer or HPLC analysis providing the similar result. The concentration of loaded DOX on apatite-DOX particles were 0.56±0.11 µM, 0.51±0.17 µM when 20 µM DOX, 0.97±0.19 µM, 0.93±0.16 µM when 40 µM DOX, and 1.50±0.32 µM, 1.41±0.32 µM when 80 µM DOX was added during complex formation, respectively. Thus, DOX incorporation increased with higher DOX concentration added during particle formation and maximum 2.785 mol% DOX was incorporated in the apatite particles when the initial DOX was added at 20 µM during complex formation. We assume that the apatite-DOX formation is based on electrostatic interactions where the positively charged DOX binds to negatively charged carbonate or phosphate-rich domain of the apatite particle. To clarify our assumption, we measured the surface charge of complex particles and found that ζ-potential of the apatite-DOX particle was more electropositive than that of only apatite particle. Moreover, ζ-potential of the apatite-DOX particle with higher DOX loading was more electropositive than that of complex particle with lower loading (data not shown here). The positively charged DOX could electro-statically bind with the negative ions like carbonate and phosphate in apatite during complex formation, making the net charge of the complex more electropositive than that of the apatite only.

### Characterization of generated particles by ATR-FTIR spectroscopy

**Figure 1:**
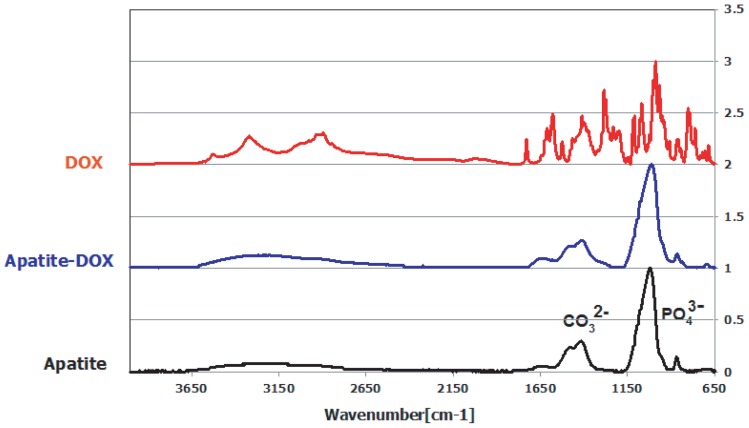
The FTIR spectrum of pure DOX, apatite and apatite-DOX complexes. After generation of apatite-DOX nanopaticles, the centrifuged pellet of the apatite particles was repeatedly washed by ddH_2_O and then lyophilized by freeze drying and checked the spectrum under FT/IR-230, JASCO.

We then characterized the lyophilized apatite-DOX conjugate particles by ATR-FTIR spectroscopy and found that the FTIR spectrum of apatite-DOX was similar to that of apatite particles. The FTIR spectra revealed the formation of carbonate apatite particles by identifying carbonate in the apatite material, as evident from the peaks between 1410 and 1540 cm^−1^ and at approximately 880 cm^−1,^ as well as phosphate, as shown by the peaks at 1000–1100 and 550–650 cm^−1^ (Figure 1). However, it was interesting that the FTIR spectrum of apatite-DOX showed stronger absorbance peaks at 650, 1250, 1650 and 3250 cm^−1^ than that of apatite particles. The absorbance peaks in 1250 and 3250 cm^−1^ were shifted a little bit due to hydrogen bonding to the carbonate and hydroxyl group respectively. Contrary to the apatite particles, both the FTIR spectrum of apatite-DOX and only DOX showed absorbance peak at 850 cm^−1^ with slight shifting (Figure 1). This data clearly revealed the direct evidence of DOX binding with carbonate apatite particles.

### Size determination of apatite-DOX complexes by AFM, DLS and TEM

**Figure 2:**
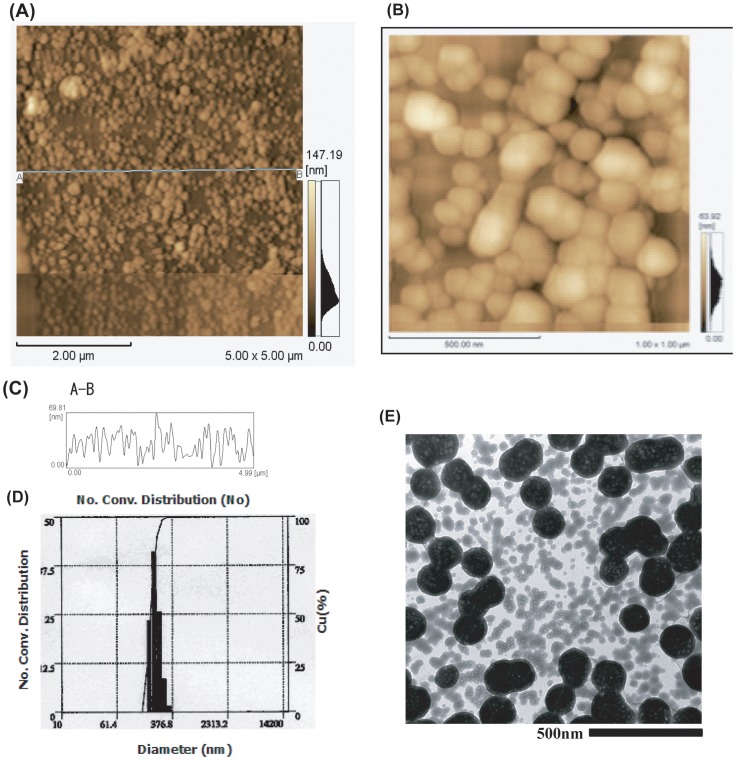
Morphology and size distribution of apatite-DOX complexes. Different techniques like A–C) AFM, D) DLS, and E) TEM were applied to characterize the nanoparticles. AFM study revealed that the maximum size was 69.8 nm. The cross-sectional curve profile was drawn between A and B as shown in figure A) where as B) shows the enlarged image focusing on the central area showed single spherical particles as well as the combined bodies consisting of several single particles and C) reveals the height distribution of nanoparticles obtained from the cross-sectional curve between A and B.

Since size of the carrier predominantly affects the overall cellular uptake through endocytosis and it is evident that endocytosis of a large complex is usually less efficient than that of a small one [31], therefore, the size distribution of apatite-DOX complexes was measured using different techniques. Detailed information on particle size was obtained by AFM (Figure 2A). Enlarged image focusing on the central area showed single spherical particles ranging from 30 to 50 nm as well as the combined bodies consisting of several single particles (Figure 2B). The maximum height of individual single particle of carbonate apatite was found to be 69.8 nm when the cross-sectional curve profile was drawn between the two arbitrary points (Figure 2C). The average diameter of the particles was estimated at 290.6 nm as determined by DLS (Figure 2D). From AFM observation, the relatively small-size particles constitute the majority of the total particles and accounts for the maximum amount of the whole particle weight. This suggests that the majority of the apatite-DOX complexes would be able to be efficiently endocytosed by cells [31]. The morphological observation of apatite-DOX complexes by TEM revealed that the particles are compact and spherical shapes with particle sizes being 100–200 nm (Figure 2E). The size distribution analyses by TEM and AFM suggest that the size of a single apatite-DOX particle was 70–80 nm and they tend to adhere, making up to 200∼300 nm as revealed by dynamic light scattering method. In addition, we found that the Ca^2+^ concentration prior to the formation of apatite-DOX particles could control the particle size. Since the large size impedes rapid internalization and small size suffers from insufficient loading of drugs [16], the appropriate particle size is certainly important. The Ca^2+^-mediated manipulation of the nanoparticle size may also confer the advantage that apatite-DOX could go out of tumor vessels but not from normal vessels and facilitate passive targeting toward the tumor by EPR effect [34].

### Observation of uptake behaviour of apatite-DOX nanoparticles

**Figure 3:**
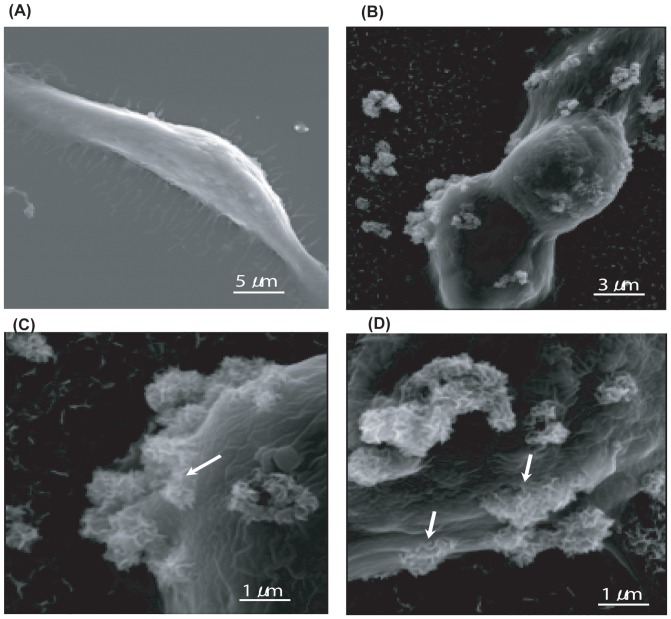
Observation of cellular uptake behavior of carbonate apatite by SEM. Scanning electron microscopy revealed a process of uptake behavior into HCT116 colon cancer cells. A) The control HCT116 devoid of apatite particles. B) The particles attached on the cell. C) The particles were getting internalized at as early as 45 min, and D) further going into the cells at 90 min.

In order to evaluate the process of uptake behavior of carbonate apatite nanoparticles *in vitro*, we performed the scanning electron microscopy after delivery onto HCT116 colon cancer cells. As shown in Figure 3, comparing with the control, devoid of apatite particles (Figure 3A), the particles are attached on the cell surface and getting internalized at as early as 45 min (Figure 3B and 3C) after delivery, consequently going further into the cells at 90 min (Figure 3D). One advantage of carbonate apatite carrier is that it provides huge uptake of drugs into the tumor cells through efficient endocytosis, as visualized by SEM.

### Cellular uptake study and quantitative uptake measurement by flow cytometry

**Figure 4:**
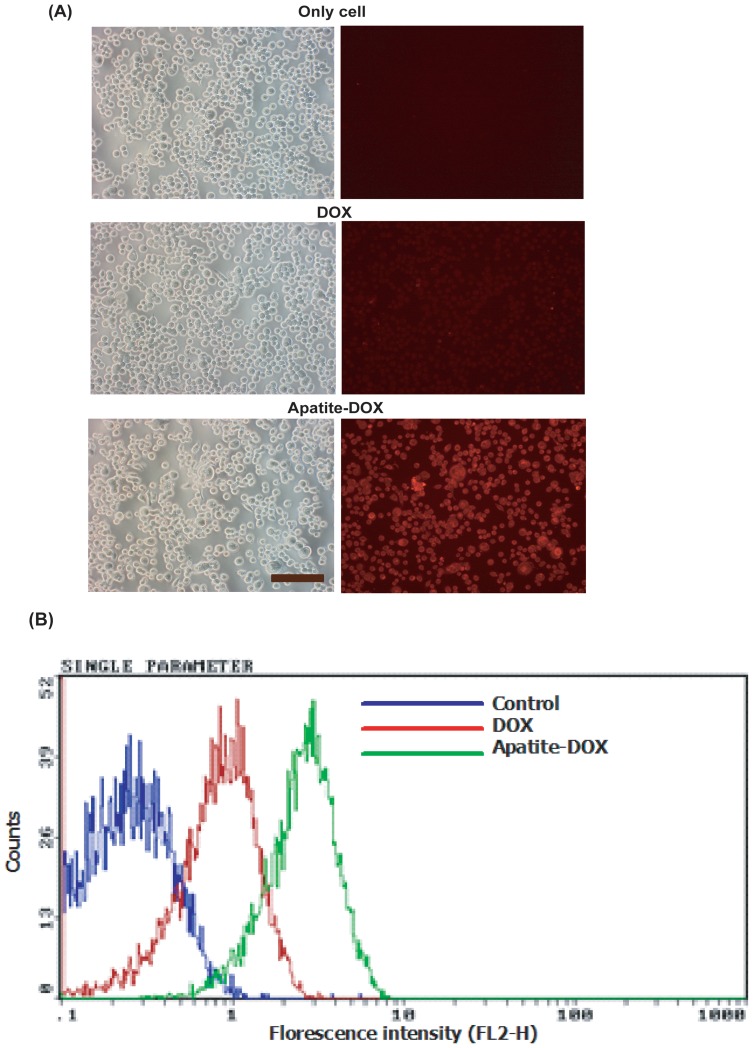
Analysis of DOX uptake by fluorescence microscopy and flow cytometry. A) Observation of cellular uptake of apatite-DOX by fluorescence microscopy in SW480 cells. Cells were grown (5×10^5^ cells/dish) on 60 mm culture dishes. After 1 day, the medium was replaced by 1 ml of fresh medium and free DOX or DOX-loaded apatite particles (200 nM DOX equivalents) were added. After 4 h, the culture dishes were washed 3 times with PBS and the extracellularly bound particles were removed by 5 mM EDTA in PBS. Scale bar, 200 μm. B) Analysis of cellular uptake of apatite-DOX by flow cytometry in NIH:OVCAR-3 cell line. The cells were seeded (0.5×10^6^cells/dish) in a 60 mm tissue culture dish, incubated overnight. 5 ml of DOX-loaded particles prepared with 200 nM DOX concentration in medium was introduced to each well and incubated for 4 h. The cells were then trypsinized, washed three times with PBS solution and then fixed with 10% formaldehyde. After filtering through a nylon mesh, cell fluorescence was measured by flow cytometry.

Both therapeutic binding to the particles and particle size contribute to the overall uptake of therapeutics by cells. Therefore, Free DOX or apatite-DOX (200 nM equivalent) complexes were subsequently incubated with cancer cells in order to investigate whether the apatite-DOX complexes could effectively be internalized by cells and cellular fluorescence was observed by a fluorescence microscope following 4 h incubation and removal of the extracellular particles by EDTA. As shown in Figure 4A, free DOX were found inefficient to enter freely into SW480 cell lines whereas apatite – DOX particles displayed an enhanced cellular delivery of DOX at 4 h.

Flow cytometry has been used for quantitative determination of DOX uptake in cells. Indeed, since DOX itself is fluorescent, it was used directly to measure cellular uptake without additional markers where fluorescence intensity should be directly proportional to the amount of DOX internalized. After delivery and incubation for 4 h, the cells (NIH:OVCAR3) were trypsinized, washed three times with PBS solution and then fixed with 10% formaldehyde. After filtering through a nylon mesh, cell fluorescence was measured by flow cytometry. Flow cytometry analysis showed that 92% of NIH:OVCAR3 cells were found to be florescent positive at 4 hr following delivery of apatite-DOX nanoparticles, while only 21% cells were florescent positive with the same dose of free drug (200 nM) (Figure 4B). The quantitative assay indicates that cellular uptake of DOX is at least imes more efficient for carbonate apatite than in case of free DOX (Figure 4B). As shown in Figure 4A and B, while free DOX uptake by the cells was low, carbonate apatite particles enhanced cellular DOX delivery to a significant extent probably due to the strong affinity of DOX to the apatite particles and the small sizes of the resulting complexes leading to the efficient endocytosis.

### Effect of crystal dissolution of apatite-DOX complexes in endosomal acidic pH condition

**Figure 5:**
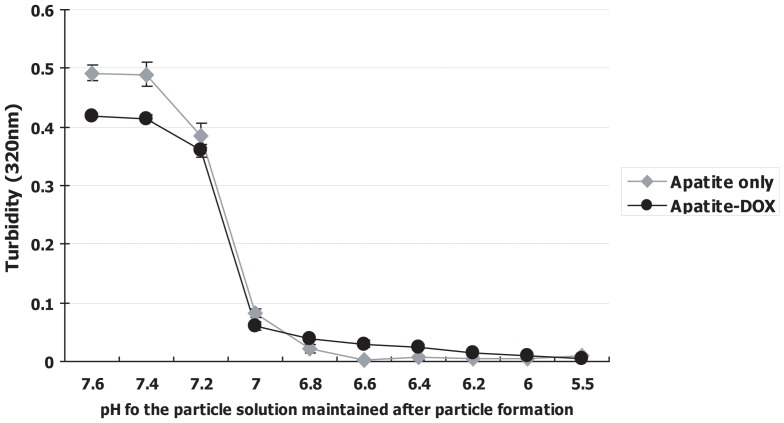
Dissolution profiles of apatite-DOX particles under different pH conditions. Optical density of the suspension of apatite-DOX complexes was plotted against pH value. Data represent mean value ± SE (n = 3).

One of the major challenges for a delivery system is its capacity for the fast release of the associated drugs after cellular internalization. The release of the DOX from the apatite carrier is essentially required for binding with DNA for its cell killing mechanism just after cellular uptake of apatite-DOX complexes. Particles supposed to be capable of releasing associated drug following endocytosis if they can rapidly be dissolved in endosomal acidic pH. We, therefore, checked the effect of low pH mimicking the endosomal pH on the dissolution of apatite-DOX particles and turbidity (320 nm) measurement was done as an indicator of their solubilization, following an acid load in a solution of generated apatite particles. The optical density of apatite-DOX complexes and apatite particles themselves act as an indicator of particle existence with lower turbidity indicating higher dissolution due to the existence of fewer no. of particles and with decreasing pH from 7.5 to 6.8, nanoparticles were completely solubilized within 1 min (Figure 5). These results indicate that the optical density of apatite-DOX complexes and apatite themselves rapidly decreased with a decrease of pH, suggesting that the apatite-DOX complexes were almost completely dissolved in the condition below pH 6.6, which might contribute to the destabilization of endosomes following endocytosis of apatite-DOX complexes, resulting in the release of DOX to the cytoplasm [24], [30]–[31].

### *In vitro* pH-responsive drug release behaviors

In addition to the enhancement of drug efficiency, the elimination of toxic side effects of drugs, especially poorly water-soluble drugs, is also vitally important and significant to anti-cancer therapy and overcoming of MDR. One of the widely accepted routes is to endow nano drug delivery systems with the pH-responsive drug release character [35]–[38]. The perfect case is that drugs do not or hardly release in normal tissues and blood (pH∼7.4), but can responsively release in tumor tissues, or even within cancer cells, to selectively kill the cancer cells (pH  = 4∼6.8). Though some drug delivery systems have been designed to release drugs under *in vitro* simulated acidic conditions [36]–[38], however, to effectively suppress drug release as slowly as possible at normal physiological conditions pH (pH 7.4) is still a great challenge, especially under *in vivo* conditions. Fortunately, we discover that the designed Apatite-DOX nanoparticle has such a desired pH-responsive drug release feature.

**Figure 6:**
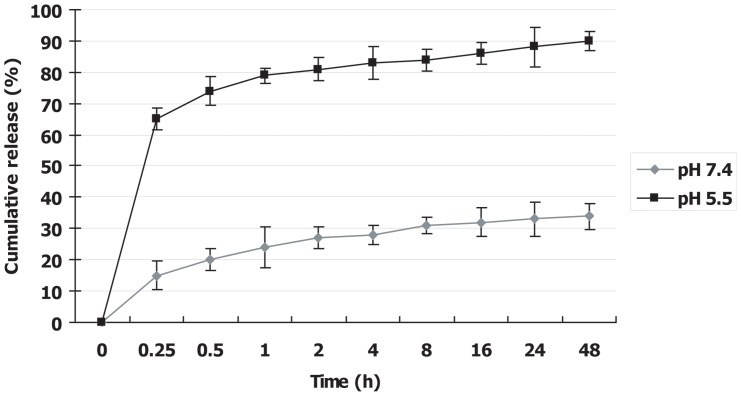
*In vitro* pH-responsive drug release behaviors of apatite-DOX nanoparticles. The release media of different pH values which were used to simulate the alkalescent conditions in normal tissues and blood (pH∼7.4) and the acidic conditions in tumor (pH = 4∼6.8). The nano apatite-DOX hardly released DOX in the pH∼7.4 release medium (PBS), but responsively released DOX in pH 5.5 acidic media (sodium acetate). Data represent mean value ± SE (n = 3).

From the drug release curve (Figure 6), it is clear that a very small amount of DOX is released from carbonate apatite nanoparticles in a very slower fashion at pH 7.4 in PBS simulating normal physiological conditions, and only less than 35% of DOX was released after immersion for as long as 48 h, which is indeed an extraordinarily low drug-released profile in such a long release time period. When the pH values of release media decreased from 7.4 to 5.5 for simulating cancer conditions, the DOX release rates became remarkably faster with the initial burst of 65% release only within 15 min, indicating that DOX-loaded carbonate apatite nanoparticles were rapidly dissolved and released drug in weakly acidic environments. After immersion for 48 h, the DOX-released percentage could reach about 90% in pH 5.5 releases medium. The pH-triggered drug release from carbonate apatite nanoparticles, in which the DOX was bound with the apatite under normal physiological conditions and released at reduced pH typical of micro-environments of intracellular lysosomes or endosomes or cancerous tissue, more generally, provides an in-built mechanism for selective drug release for the intracellular and *in vivo* evaluation.

### Apatite-DOX mediated cytotoxicity in tumor cell lines

**Figure 7:**
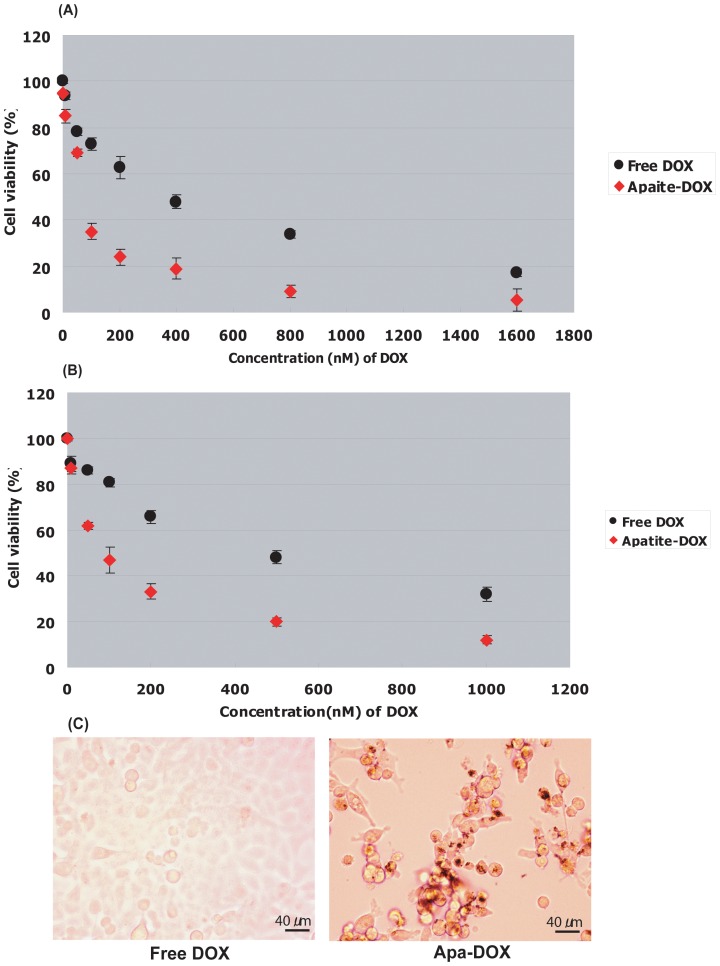
*In vitro* cytotoxic effect of DOX and apatite-DOX. The cytotoxicity in response to different concentrations of free and apatite loaded drugs towards A) SW480 colon carcinoma cells, B) ovarian carcinoma cells NIH:OVCAR-3 estimated by MTT cell proliferation assay. Data represent mean value ± SE (n = 3). C) Microscopic observation on HCT116 colon cancer cells after 72 h (DOX dose: 150 nM). Magnifications: x50.

We performed a series of *in vitro* cytotoxicity assays to evaluate the anti-cancer potential of nanoparticle complex of apatite-DOX, using colon cancer cells like SW480, HCT116 and ovarian cancer cell like NIH:OVCAR-3 for 0∼72 h and compared its efficacy to that of conventional free DOX. The IC_50_ of free DOX was 370 nM and 490 nM whereas IC_50_ of apatite-DOX nanoparticle was only 75 nM and 80 nM after 72 hr in SW480 (Figure 7A) and NIH:OVCAR-3 cell lines (Figure 7B), respectively. Apatite-DOX achieved almost 5–6 times more cytotoxicity in those cell lines than that of free drug. Moreover, enhanced growth inhibitory effects were also observed in HCT116 colon cancer cells (IC_50_: 194 nM for free DOX, and 93.5 nM for Apatite-DOX, data not shown). The profound cell killing effects of apatite-DOX compared to free DOX were observed under the optical microscope in HCT116 colon cancer cells (Figure 7C). *In vitro* culture studies showed a marked cell killing ability by apatite-DOX nanoparticles in 3 carcinoma cells tested in comparison with free DOX. The superb effects provided by the delivery system could be explained through two steps. One advantage of carbonate apatite carrier is that it provides huge uptake of drugs into the tumor cells through efficient endocytosis, as visualized by scanning electron microscopy. After cellular internalization, apatite-DOX particles could be quickly degraded in response to low pH in the acidic endosomes or lysosomes, as suggested by dissolution assay where we found that apatite-DOX particles were highly stable at physiological pH 7.4 but dissolved at endosomal pH 5.5. The efficient uptake and quick release of DOX from the particles were consistent with our findings of DOX distribution as assayed by fluorescent microscopy and flow cytometry. Moreover, the dissolution and release profile indicated that apatite-DOX nanoparticles might be a useful tool both for tumor extracellular and intracellular pH targeting [39]–[41] to overcome MDR [42]. To tackle the multifaceted MDR mechanisms, nowadays it is hypothesized that a focal high dose strategy works at cellular level rather than at systemic level. Thus, a local high dose might overwhelm most resistant mechanisms limiting their intrinsic defense capability because even extremely resistant experimental MDR cells could be killed at high drug concentrations [43]–[48].

### *In vivo* antitumor activity mediated by apatite-DOX complex nanoparticles

**Figure 8:**
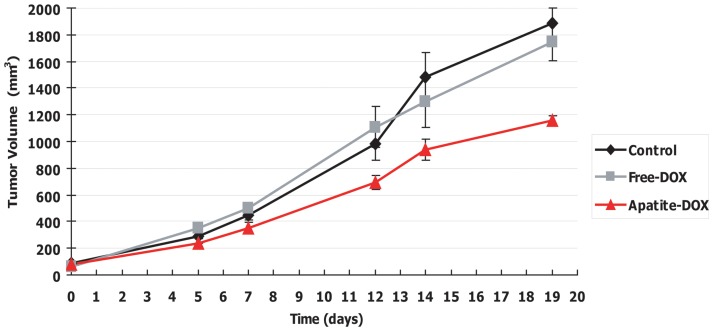
Inhibition of colorectal tumor growth by the carbonate apatite-mediated doxorubicin delivery. HCT116 cells were inoculated s.c. in the both left and right flanks of the mice (n = 6). When the diameter of tumors reached φ5 mm, Free-DOX at dose of 0.33 mg/kg/day or Apatite-DOX containing the same amount of DOX were administered by i.v. injection into the tail vein on days 0,1,2,7,8,9,14,15, and 16. Saline was administrated in case of control animals. Significant differences in the tumor volume were found between Control or Free-DOX and Apatite-DOX on day 12 and 19 (p<0.05). Data represent mean value ± SE (n = 6).

The *in vivo* anti-tumor efficacy of free DOX and DOX-loaded pH-responsive carbonate apatite nanoparticles was evaluated on colorectal tumor model in BALB/cA nude mice. The tumor growth rates of mice treated with saline and carbonate apatite (without DOX) were similar; indicating that pH responsive carbonate apatite alone had no effect on tumor growth (data not shown). To evaluate the advantage of carbonate apatite as drug delivery carrier, we set the dose of DOX as 0.33 mg/kg/day, which is not strong enough to be effective. When the mice were treated with free DOX or apatite-DOX, free-DOX did not show the significant tumor growth inhibition as compared to control group. However, from day 5, apatite-DOX began to exhibit moderate response to the tumor. As shown in Figure 8, on both day 12 and 19, the significant tumor inhibition was observed between the groups treated with apatite-DOX and free-DOX (p<0.05). In spite of non-effectively low dose of DOX, apatite-DOX had higher antitumor activity than free-DOX, which is in agreement with our *in vitro* finding. This enhanced tumor inhibitory effect might be due to the huge uptake of drugs through efficient endocytosis of apatite-DOX nanoparticles followed by the quick release of drugs owing to the particle dissolution at endosomal low pH, which could avoid extra- and intra-cellular drug inactivation and drug resistance usually observed for passive diffusion-mediated drug entry, thus executing superb *in vitro* cancer cell proliferation inhibition. Other pH-sensitive approaches to the delivery of anti-tumor drugs, which also possessed the pH-triggered drug release features, however, are not suitable due to their requirements of too low pH for drug release. More effective drug delivery requires a more prompt response to a small pH change, as in the weakly acidic extracellular and intracellular pH environments of tumor tissues. Our pH-responsive carbonate apatite nanoparticles containing DOX showed huge uptake of DOX and fast drug release at weakly acidic pH within 6 h, indicating that these nanoparticles are more suitable for anticancer drug release at tumor sites. Also, the pH-responsive carbonate apatite nanoparticles were stable under physiological conditions, with the drug release being minimized; indicating that this apatite-based drug delivery system could reduce unwanted side effects of anticancer drugs during cancer therapy.

## Conclusion

In the current study, we successfully fabricated a nano size delivery device for anticancer drug DOX into tumor cells using the inorganic crystals of carbonate apatite having characteristics of nano-scale and quick release of DOX in response to endosomal low pH, resulting in increased cellular uptake and significantly enhanced colon tumor inhibitory effects even at a very low dose of anticancer drug. The extraordinary proliferation inhibitory effect in the tumor cell lines was achieved by the virtue of its pH sensitivity resulting in quick release of the drugs from the carrier, thus achieving significant antitumor activity in colorectal tumor model in mice. Therefore, using the carbonate apatite and a chemotherapeutic agent, we propose a novel nano-based pH targeting therapeutic strategy against human malignancies, which is highly promising for cancer therapy.
